# Prevalence of Functional Constipation and Relationship with Dietary Habits in 3- to 8-Year-Old Children in Japan

**DOI:** 10.1155/2018/3108021

**Published:** 2018-02-27

**Authors:** Asami Fujitani, Tsuyoshi Sogo, Ayano Inui, Kiyoshi Kawakubo

**Affiliations:** ^1^Kanagawa University of Human Services Faculty of Health & Social Services School of Nutrition & Dietetics, 1-10-1 Heisei-cho, Yokosuka City, Kanagawa Prefecture 238-8522, Japan; ^2^Department of Nutrition and Diet, Saiseikai Yokohama City Tobu Hospital, 3-6-1 Shimosueyoshi, Tsurumi-ku, Yokohama, Kanagawa Prefecture 230-0012, Japan; ^3^Department of Pediatric Hepatology and Gastroenterology, Saiseikai Yokohama City Tobu Hospital, 3-6-1 Shimosueyoshi, Tsurumi-ku, Yokohama, Kanagawa Prefecture 230-0012, Japan; ^4^Department of Food Science and Nutrition, Faculty of Home Economics, Kyoritsu Women's University, 2-2-1 Hitotsubashi, Chiyoda-ku, Tokyo 100-0003, Japan

## Abstract

**Objectives:**

To determine the prevalence and effect of dietary habits on functional constipation in preschool and early elementary school children in Japan.

**Study Design:**

A total of 3595 children aged 3 to 8 years from 28 nursery schools and 22 elementary schools in Yokohama City, Kanagawa Prefecture, Japan, were evaluated. The subjects were divided into a functional constipation group and a nonfunctional constipation group according to the Rome III criteria. Dietary intake data were collected using a brief-type, self-administered, diet-history questionnaire validated for Japanese preschool-aged children.

**Results:**

Of the 3595 subjects evaluated, 718 (20.0%) had functional constipation. The association between functional constipation and gender was not statistically significant (*p* = 0.617). A decrease in bowel frequency was observed in 15.9% of those with functional constipation. There was no significant difference in the proportion of participants in the constipation group by age (*p* = 0.112). Binomial logistic regression analysis indicated that only fat per 100 kcal positively correlated with functional constipation [odds ratio = 1.216, 95% confidence interval: 1.0476–1.412].

**Conclusions:**

Functional constipation is common among children in preschool and early elementary school in urban areas of Japan. Parents should pay attention to constipation-related symptoms other than defecation frequency. A high-fat diet should be avoided to prevent functional constipation.

## 1. Introduction

Constipation is one of the most common health problems in children and adults. Variation in the normal bowel habit and symptoms recognized as constipation has made it difficult to accurately diagnose the condition in children. In addition, overflow incontinence and encopresis, both symptoms of functional constipation (FC), are often considered simply as problematic bowel habits and not as symptoms of FC. Therefore, it is difficult to clarify the current state of FC in childhood. The Rome III criteria [1, 2] have been widely applied for the diagnosis of functional gastrointestinal disorders, including FC, and have been commonly used in clinical research. The Rome III criteria include measures of defecation frequency, stool consistency, and other symptoms of FC. However, there are few reports on the prevalence of FC in children in East Asia based on the Rome III diagnostic criteria [3–5].

It is well known that dietary habit can affect defecation, and as such, regional disparity should be considered when determining the prevalence of constipation. Although increasing the intake of dietary fibre and water is often recommended as a dietary therapy for patients with constipation, the influence of total calories and individual nutrients on FC in childhood have not been clarified. A brief-type, self-administered, diet-history questionnaire (BDHQ) developed by Kobayashi et al. has been validated for dietary intake assessment in adults [6]. Asakura et al. revised the BDHQ for use in children aged 3–6 years (BDHQ3y) and then validated the revised questionnaire in Japanese children [7]. This study was aimed at determining the prevalence of FC in Japanese children aged 3–8 years using the Rome III criteria and its relationship with their dietary habits using BDHQ3y.

## 2. Materials and Methods

### 2.1. Study Setting and Participants (Figure 1)

Two types of questionnaire were distributed to the guardians of 2052 children attending 28 public or private nursery schools and 4451 children attending 1st or 2nd grade at 22 public elementary schools (6503 people in all) in Tsurumi Ward, Yokohama City, Kanagawa Prefecture, Japan, in September 2013. The questionnaires were collected between October and November 2013. One questionnaire assessed defecation and physical status, and the other was the BDHQ3y, which assessed total calories consumed and the intake of individual nutrients. The questionnaires were enclosed and concealed in envelopes by each guardian. The envelopes were collected at each school and sent back to the authors by a representative at each school.

This survey included questions on sex, age, height, weight, defecation frequency, fecal incontinence, withholding behaviour, painful defecation, stool consistency, and the presence of large-diameter stools. Guardians were required to answer questions regarding defecation frequency, stool consistency using the Bristol Stool Scale type, and frequency at 4 grades (always, sometimes, never, and hard to say) for other details related to defecation. Guardians had to choose the Bristol Stool Scale type that best represented their children's stools using a picture chart. The picture chart was accompanied by descriptors that had been translated into Japanese.

The BDHQ3y is a four-page structured questionnaire that asks about the consumption frequency of selected foods commonly eaten in Japan, general dietary behaviour, and usual cooking methods. Estimates of the daily intake of different foods (66 items in total), total calories, and selected nutrients were calculated using an ad hoc computer algorithm for the BDHQ3y based on the Standard Tables of Food Composition in Japan [8]. Although the BDHQ3y has been validated, the validation study was conducted in children aged 3 to 4 years. As the current study included subjects aged 5–8 years, it is possible that adjustments for age in the nutritional value calculations did not directly represent actual changes in dietary intake.

### 2.2. Classification of Defecation Status

The defecation status of each subject was classified according to the Rome III criteria (Table 1). Questions about defecation in the questionnaire were consistent with the items in the Rome III criteria for FC. Questions answered as “always” or “sometimes” were judged as positive. The fourth item in the Rome III criteria, which asks about painful defecation or stool consistency, was judged as positive if a subject always or sometimes had painful defecation or always had hard stool of Bristol Stool Scale type 1 or 2. The fifth item in the Rome III criteria, which asks about the presence of a large fecal mass in the rectum, was omitted because this item as well as the exclusion of irritable bowel syndrome requires a physician for the answer. Subjects with 2 or more positive items were classified into the FC group, and the remaining participants were classified into the non-FC group.

### 2.3. Assessment of Physical Status

Height, body weight, and body mass index (BMI) were evaluated based on the report of a national growth survey in preschool children [9] and an annual report of school health statistics research published in 2000 [10], which has been widely used to assess the physical status of children in Japan. BMI was calculated as body weight in kilograms divided by height squared in meters (kg/m^2^). Height and weight were evaluated using *z*-scores (standard deviation (SD) scores). The *z*-score is the deviation of an individual's value from the median value of a reference population, divided by the standard deviation of the reference population (or transformed to normal distribution). The *z*-scores (SD scores) for height and weight were calculated according to the standard formula *z* = (sample data − mean)/SD. *z*-Scores for height and weight and the percentile of BMI were calculated using the taikakushisu_v3.xlsx spreadsheet (http://jspe.umin.jp/medical/files/taikakushisu_v3.xlsx) developed by the Standard Value Committee of The Japanese Society of Pediatric Endocrinology/The Japanese Association for Human Auxology.

### 2.4. Nutritional Assessment

The BDHQ3y calculates the intake amounts of 99 different types of food nutrients, including total calories, meal weight, water, protein, fat, carbohydrates, 15 food groups, and 78 ingredients based on an assessment of food-intake frequency per month. Inputting questionnaire data and conversion of the quantities of calories, nutrients, and food groups consumed were performed by the developer's company (EBNJAPAN, Tokyo), and we then used the converted data for analysis. We analysed 15 items, including total amount per day of meal weight, total calories, water content in foods, protein, fat, carbohydrates, calcium, magnesium, phosphorus, soluble dietary fibre, insoluble dietary fibre, total dietary fibre, sodium chloride, juice, and water including tea. Twelve items, excluding juice and water, were analysed as the nutritional equivalent per 100 kcal.

### 2.5. Ethical Considerations

This survey was approved by the Ethics Committee of Saiseikai Yokohama City Tobu Hospital (protocol number 201230). Response to the questionnaire was regarded as an agreement to participate, in accordance with Ethical Guidelines for Epidemiological Research published by the Ministry of Health, Labor and Welfare.

### 2.6. Statistical Analysis

The prevalence of FC and the symptoms detailed in the Rome III criteria were examined using Pearson's *χ*^2^ test. Associations between FC and physical status, such as height and weight and energy and nutrient intake, were initially examined using *t*-tests, and analysis of covariance was performed with age as a covariate to compare the FC group and non-FC group. Then, binomial logistic regression analysis was performed to identify factors independently associated with FC in children. All statistical analyses were performed using SPSS statistical software (version 23, IBM Japan). Two-sided *p* values < 0.05 were considered statistically significant.

## 3. Results

### 3.1. Background and Physical Status

A total of 3932 guardians returned the questionnaires which gave a collection rate of 58.7%. Overall, 3643 subjects answered both questionnaires. Of these, 14 subjects aged 9 years, 3 subjects who did not give their age, and 31 subjects who gave incomplete descriptions were excluded from further analysis. Thus, in total, 3595 subjects were analysed in this study. The characteristics of the analysed children (*n* = 3595) are shown in Table 2. Among the 3595 subjects, 718 were classified into the FC group (20.0%). There was no statistically significant difference either in the male-to-female ratio (*p* = 0.617) or in age (*p* = 0.112) between the two groups. However, the average age of the girls in the FC group was significantly higher than that of the girls in the non-FC group (6.6 ± 1.3 years and 6.5 ± 1.3 years, resp., *p* = 0.015). The mean body weight and the mean BMI were higher in the FC group than the non-FC group (*p* = 0.047 and *p* = 0.049, resp.), but in the analysis using age as a covariate, only the mean BMI percentile value was significantly higher in the FC group than the non-FC group (*p* = 0.042). Numbers of obesity (BMI percentile > 90) were 59 (8.2%) in the FC group and 186 (6.5%) in the non-FC group, but there was no significant difference between the two groups (*p* = 0.059). There was no significant difference in the proportion of children in the constipation group by age (*p* = 0.290). Binomial logistic regression analysis did not identify any factors associated with FC.

### 3.2. Rome III Criteria (Table 3)

Among the six items included in the Rome III criteria, “history of excessive stool retention” and “history of painful bowel movements” were frequently observed in the FC group (73.6% and 61.5%, resp.), with odds ratios as high as 24.5 and 11.0, respectively, and high sensitivity and specificity. The incidences of the items “≤2 defecations per week” and “history of large-diameter stools that obstruct the toilet” in the FC group were low (15.9% and 21.0%, resp.), although the specificity for both was 0.98.

### 3.3. Nutritional Assessment

The FC group had significantly greater fat intake than the non-FC group (45.9 ± 13.6 g and 44.4 ± 12.9 g, resp., *p* = 0.005) and greater juice intake (94.2 ± 127.5 g and 80.9 ± 98.8 g, resp., *p* = 0.009) but less intake of water, including tea (301.6 ± 168.0 g and 318.2 ± 168.4 g, resp., *p* = 0.018) (Table 4). Similar results were obtained from an analysis using age as a covariate. No significant differences were found in the intake of dietary fibre (insoluble dietary fibre or water-soluble dietary fibre) between the two groups. Comparing the nutritional equivalents, defined as the intake of each nutrient per 100 kcal of ingested energy, between the FC group and the non-FC group revealed that the FC group had significantly lower total meal weight (114.7 ± 20.6 g and 117.3 ± 21.0 g, resp., *p* = 0.003), meal water content (93.0 ± 20.4 g and 95.5 ± 20.9 g, resp., *p* = 0.004), magnesium intake (12.5 ± 2.0 mg and 12.8 ± 2.0 mg, resp., *p* = 0.002), soluble dietary fibre intake (134.0 ± 3.6 mg and 138.7 ± 3.7 mg, resp., *p* = 0.003), insoluble dietary fibre intake (424.5 ± 9.1 mg and 442.3 ± 9.9 mg, resp., *p* < 0.001), total dietary fibre intake (570.3 ± 128.8 mg and 593.5 ± 138.4 mg, resp., *p* < 0.001), and sodium chloride intake (667.0 ± 137.9 mg and 680.7 ± 136.2 mg, resp., *p* = 0.016) than the non-FC group. In contrast, the intake of fat was significantly higher in the FC group than in the non-FC group (3.3 ± 0.6 g and 3.2 ± 0.6 g, resp., *p* = 0.019). Binomial logistic regression analysis indicated that only fat per 100 kcal positively correlated with FC [odds ratio = 1.216, 95% confidence interval: 1.0476–1.412] (Table 5).

## 4. Discussion

We could analyse 25.4% of the population of children aged 3 to 8 years in the survey area. Hence, our results reflect the prevalence of FC among children aged 3 to 8 years living in urban areas in Japan, which is approximately 20% regardless of age or sex. It should be noted that because the item in the Rome III criteria that asks about the presence of a large fecal mass in the rectum was not included in the questionnaires in this survey, the prevalence of childhood FC may be higher.

Asakura et al. conducted a school-based survey to examine the relationship between constipation and lifestyle factors, including dietary intake, among preschool-aged children in Japan [11]. The participants were recruited from 44 of 47 prefectures in Japan, and 5309 children were analysed. They considered children with three or fewer bowel movements per week to have constipation, and they did not ask about the other items found in the Rome III criteria for FC. Their results showed that 8.4% of preschool-aged children had constipation, which is likely lower than the actual number because their criteria ignored important symptoms of FC such as fecal incontinence, stool retention, painful or hard bowel movements, and large-diameter stools. Using the same questionnaires about bowel habits as those used in the present study, we previously surveyed 643 children aged 3 to 9 years in Sasayama City, Hyogo Prefecture, which was 26.3% of this subpopulation in the area, and calculated an FC prevalence of 14.6% [12]. The difference in the prevalence of FC between Sasayama City and Yokohama City is likely due to regional differences because Sasayama City is in a rural area of Japan. In other school-based studies using the Rome III Criteria, the prevalence of FC ranged from 10 to 15.9%, although these studies evaluated children who were 8 years of age or older [13–18]. A systematic review performed by van den Berg et al. showed that the prevalence of childhood constipation in the general population ranged from 0.7% to 29.6% and the age group in which constipation is most common varied between studies [19]. The present work is the largest school-based study to evaluate the prevalence of FC in children aged 3 to 8 years.

In this study, a history of excessive stool retention and painful bowel movements were observed in 73.6% and 61.5% of the children in the FC group. In contrast, only 15.9% of the children in the FC group experienced 2 or less defecations per week. In previous studies, such differences between items in the Rome III criteria were not observed [4, 20]. In our previous study conducted in Sasayama City, a decreased frequency of defecation was not common in children classified as having FC [12]. This unexpected finding might be a result of the specific questionnaire used. However, parents may not notice that a child is constipated if there is no decrease in defecation frequency. Moreover, it has been shown that children who begin treatment at the age of 2 years or younger have a better prognosis for FC than children who begin treatment over the age of 2 years [21]. It has also been shown that poor clinical outcomes in adulthood are associated with an older age at onset, a longer delay between onset and first visit to an outpatient clinic, and a low defecation frequency [22]. Our results indicate that constipation symptoms other than the frequency of bowel movements should be evaluated in children and that treatment for constipation should be initiated before the age of 3.

In this study, univariate analysis revealed that water intake, meal weight/100 kcal, water content per meal/100 kcal, magnesium intake/100 kcal, dietary fibre intake/100 kcal, and sodium chloride intake/100 kcal were all significantly lower in the FC group compared to the non-FC group, whereas the intake of juice was significantly higher. However, binomial logistic regression analysis showed that only fat per 100 kcal positively correlated with FC [odds ratio = 1.216, 95% confidence interval: 1.0476–1.412]. In mouse experiments, feeding of a high-fat diet resulted in intestinal dysbiosis and delayed colonic motility [23]. However, in human studies, a high-fat diet was not associated with retardation of colonic transit [24], although it has been shown that obesity was related to FC in children [25]. In our study, the analysis using age as a covariate showed significant difference in the mean BMI percentile value between the FC group and the non-FC group (*p* = 0.042), whereas there was no significant difference in the number of obese children between the two groups (*p* = 0.059). In normal individuals after a high-fat meal, abnormal gastrocolic reflex with prolonged retrograde phasic contraction was observed [26]. In rodent models, high-fat diet was associated with less availability of serotonin in the colon and intestinal dysbiosis which caused delayed colonic motility [23, 27]. These may explain our results even though we could not show colonic transit time in this study. In our study, binomial logistic regression analysis did not reveal any other nutrients, foods, or aspects of physical status that was associated with FC. In a previous report using the BDHQ3y, increased dietary fibre intake was significantly associated with 4 or more bowel movements per week [11]. Moreover, the intake of potatoes, pulses, vegetables, and fruits decreased the incidence of children with less than 3 bowel movements per week, whereas higher rice intake was significantly and independently associated with an increased incidence of children with less than 3 bowel movements per week. However, the Rome III criteria were not used for the diagnosis of constipation, and constipation symptoms other than the decreased frequency of bowel movements were not evaluated in the report.

The main limitations of this study were that the Japanese questionnaire on defecation was not validated before its use and the question about the presence of large fecal mass in the rectum was omitted in the questionnaire. These factors could have influenced the prevalence of FC obtained in this study.

## 5. Conclusions

The current findings suggest that FC is common among children in preschool and early elementary school in urban areas in Japan. Decreased frequency of bowel movements is not a common symptom of FC in this population, and guardians should pay attention to other symptoms, including withholding defecation and painful defecation. Consumption of a high-fat diet should be avoided to prevent FC. A longitudinal study and an expanded study of additional age groups are also needed to determine whether FC in children aged 3 to 8 years persists into late childhood, adolescence, and adulthood.

## Figures and Tables

**Figure 1:**
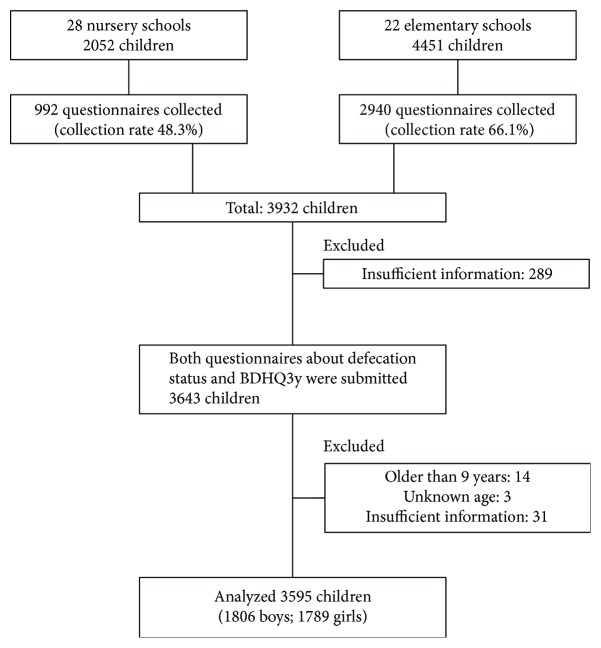
Study setting and participants.

**Table 1:** Rome III criteria for functional constipation.

<4 years	≥4 years
Must include at least 2 items for 1 month	Must include 2 or more items for at least 2 months before diagnosis
(1) Two or fewer defecations per week	(1) Two or fewer defecations in the toilet per week
(2) At least 1 episode per week of incontinence after the acquisition of toileting skills	(2) At least 1 episode of fecal incontinence per week
(3) History of excessive stool retention	(3) History of retentive posturing or excessive volitional stool retention
(4) History of painful or hard bowel movements	(4) History of painful or hard bowel movements
(5) Presence of a large fecal mass in the rectum	(5) Presence of a large fecal mass in the rectum
(6) History of large-diameter stools that may obstruct the toilet	(6) History of large-diameter stools that may obstruct the toilet

**Table 2:** Characteristics of the subjects.

		FC	Non-FC	*p* value	
		*n* = 718	*n* = 2877	*t*-test	ANCOVA
Boys : girls		367 : 351	1439 : 1438	0.617	

Mean age (years)	All	6.5 ± 1.3	6.4 ± 1.3	0.112	
	Boys	6.4 ± 1.4	6.4 ± 1.3	0.912	
	Girls	6.6 ± 1.3	6.5 ± 1.3	0.015	

Mean height (cm)	All	118.6 ± 10.0	118.5 ± 9.3	0.387	0.479
	Boys	118.6 ± 10.0	118.5 ± 9.3	0.863	0.362
	Girls	118.3 ± 8.8	117.7 ± 8.8	0.287	0.315

Mean weight (kg)	All	22.2 ± 4.9	21.8 ± 4.5	0.047	0.218
	Boys	22.5 ± 5.4	22.2 ± 4.6	0.149	0.133
	Girls	21.9 ± 4.3	21.6 ± 4.4	0.238	0.467

Mean BMI (kg/m^2^)	All	15.7 ± 1.9	15.5 ± 1.9	0.049	0.067
	Boys	15.8 ± 2.1	15.6 ± 1.9	0.033	0.193
	Girls	15.5 ± 1.8	15.5 ± 1.9	0.554	0.588

Mean height *z*-score	All	−0.15 ± 1.14	−0.08 ± 1.06	0.148	0.171
	Boys	−0.07 ± 1.16	−0.04 ± 1.09	0.722	0.296
	Girls	−0.22 ± 1.12	−0.11 ± 1.02	0.078	0.454

Mean weight *z*-score	All	−0.22 ± 1.03	−0.22 ± 0.99	0.911	0.806
	Boys	−0.15 ± 1.16	−0.26 ± 1.22	0.122	0.384
	Girls	−0.29 ± 1.01	−0.25 ± 0.96	0.592	0.544

Mean BMI percentile	All	45.47 ± 28.67	43.23 ± 28.11	0.057	0.042
	Boys	46.81 ± 29.42	43.91 ± 28.67	0.085	0.338
	Girls	44.06 ± 27.84	42.54 ± 27.53	0.355	0.290

Data are shown as the mean ± standard deviation. ANCOVA: analysis with age as a covariate (Bonferroni).

**Table 3:** Frequency of constipation symptoms including Rome III criteria.

	All (*n* = 3595)	FC (*n* = 718)	Non-FC (*n* = 2877)
	*n* (%)	*n* (%)	*n* (%)
≤2 defecations per week	162 (4.6)	111 (15.9)	51 (1.8)
Fecal incontinence	488 (13.7)	330 (46.3)	158 (5.5)
History of excessive stool retention	785 (23.5)	495 (73.6)	290 (10.9)
History of painful bowel movements	774 (22.7)	424 (61.5)	350 (12.8)
History of hard bowel movements	675 (19.0)	310 (43.6)	365 (12.7)
History of large-diameter stools that obstruct the toilet	206 (5.8)	149 (21.0)	57 (2.0)

**Table 4:** Relationship between functional constipation and consumption of energy and nutrients.

Mean amount of intake per day	Unit	FC *n* = 718	Non-FC *n* = 2877	*p* value	
				*t*-test	ANCOVA
*Energy and nutrients*					
Total meal weight	g	1606.3 ± 466.9	1616.2 ± 457.9	0.606	
Total calories	kcal	1421.8 ± 417.0	1395.2 ± 385.6	0.122	0.128
Water content per meal	g	1298.2 ± 393.7	1312.5 ± 390.8	0.380	0.378
Protein	g	48.4 ± 14.0	48.1 ± 13.5	0.631	0.666
Fat	g	45.9 ± 13.6	44.4 ± 12.9	0.005	0.007
Carbohydrates	g	198.9 ± 70.2	196.2 ± 65.0	0.346	0.376
Calcium	mg	529.6 ± 204.4	519.5 ± 198.1	0.272	0.269
Magnesium	mg	176.6 ± 54.3	177.3 ± 52.6	0.779	0.736
Phosphorous	mg	808.9 ± 257.2	801.7 ± 248.5	0.490	0.513
Soluble dietary fibre	g	1.88 ± 0.7	1.92 ± 0.7	0.170	0.164
Insoluble dietary fibre	g	5.97 ± 2.0	6.13 ± 2.0	0.062	0.057
Total dietary fibre	g	8.02 ± 2.8	8.22 ± 2.8	0.074	0.069
NaCl	g	9.22 ± 2.4	9.27 ± 2.4	0.614	0.605
Juice	g	94.2 ± 127.5	80.9 ± 98.8	0.009	0.002
Water including tea	g	301.6 ± 168.0	318.2 ± 168.4	0.018	0.015
*Nutritional equivalent*					
Total meal weight	g/100 kcal	114.7 ± 20.6	117.3 ± 21.0	0.003	0.004
Water content per meal	g/100 kcal	93.0 ± 20.4	95.5 ± 20.9	0.004	0.006
Protein	g/100 kcal	3.4 ± 0.5	3.5 ± 0.5	0.090	0.111
Fat	g/100 kcal	3.3 ± 0.6	3.2 ± 0.6	0.019	0.019
Carbohydrate	g/100 kcal	13.9 ± 1.6	14.0 ± 1.5	0.127	0.123
Calcium	mg/100 kcal	37.5 ± 10.9	37.3 ± 10.3	0.793	0.720
Magnesium	mg/100 kcal	12.5 ± 2.0	12.8 ± 2.0	0.002	0.003
Phosphorous	mg/100 kcal	57.3 ± 10.6	57.7 ± 9.9	0.346	0.395
Soluble dietary fibre	mg/100 kcal	134.0 ± 3.6	138.7 ± 3.7	0.003	0.003
Insoluble dietary fibre	mg/100 kcal	424.5 ± 9.1	442.3 ± 9.9	0.000	0.000
Total dietary fibre/day	mg/100 kcal	570.3 ± 128.8	593.5 ± 138.4	0.000	0.000
NaCl	mg/100 kcal	667.00 ± 137.9	680.7 ± 136.2	0.016	0.023

ANCOVA: analysis with age as a covariate (Bonferroni). Data are shown as the mean ± standard deviation.

**Table 5:** Binomial logistic regression analysis of functional constipation.

Factor	Logistic regression coefficient	Standard error	*p* value	Odd ratio (95% confidence interval)
Age	0.055	0.032	0.089	10.057 (0.992–1.126)
BMI percentile	0.003	0.001	0.063	1.003 (1.000–1.006)
Juice	0.001	0.000	0.008	1.001 (1.000–1.002)
Fat/100 kcal	0.196	0.076	0.010	1.216 (1.0476–1.412)
Total dietary fibre/day/100 kcal	0.001	0	0.001	0.999 (0.998–1.000)
